# Implication of Glutathione in the *In Vitro* Antiplasmodial Mechanism of Action of Ellagic Acid

**DOI:** 10.1371/journal.pone.0045906

**Published:** 2012-09-28

**Authors:** Patrice Njomnang Soh, Benoit Witkowski, Amandine Gales, Eric Huyghe, Antoine Berry, Bernard Pipy, Françoise Benoit-Vical

**Affiliations:** 1 CNRS, Laboratoire de Chimie de Coordination (LCC), Toulouse, France; 2 Université de Toulouse III Paul Sabatier, UPS, INPT, LCC, Toulouse, France; 3 Service de Parasitologie-Mycologie, Centre Hospitalier Universitaire de Toulouse, Université de Toulouse III Paul Sabatier, Toulouse, France; 4 Faculté de Médecine de Rangueil, Université de Toulouse III Paul Sabatier, Toulouse, France; 5 UMR 152 IRD-UPS, Université de Toulouse III Paul Sabatier, Toulouse, France; 6 Department of Urology and Andrology, Centre Hospitalier Universitaire de Toulouse, Toulouse, France; University of Valencia, Spain

## Abstract

The search for new antimalarial chemotherapy has become increasingly urgent due to parasite resistance to current drugs. Ellagic acid (EA) is a polyphenol, recently found in various plant products, that has effective antimalarial activity *in vitro* and *in vivo* without toxicity. To further understand the antimalarial mechanism of action of EA *in vitro*, we evaluated the effects of EA, ascorbic acid and N-acetyl-L-cysteine (NAC), alone and/or in combination on the production of reactive oxygen species (ROS) during the trophozoite and schizonte stages of the erythrocytic cycle of *P. falciparum*. The parasitized erythrocytes were pre-labelled with DCFDA (dichlorofluorescein diacetate). We showed that NAC had no effect on ROS production, contrary to ascorbic acid and EA, which considerably reduced ROS production. Surprisingly, EA reduced the production of the ROS with concentrations (6.6×10^−9^ − 6.6×10^−6^ M) ten-fold lower than ascorbic acid (113×10^−6^ M). Additionally, the *in vitro* drug sensitivity of EA with antioxidants showed that antiplasmodial activity is independent of the ROS production inside parasites, which was confirmed by the additive activity of EA and desferrioxamine. Finally, EA could act by reducing the glutathione content inside the *Plasmodium* parasite. This was consolidated by the decrease in the antiplasmodial efficacy of EA in the murine model *Plasmodium yoelii*- *high GSH* strain, known for its high glutathione content. Given its low toxicity and now known mechanism of action, EA appears as a promising antiplasmodial compound.

## Introduction

One of the major threats to world public health is malaria with an estimated annual mortality of nearly 1 million, of which more than 75% of the deaths are among African children [1]. The research for new drugs against malaria is urgently needed and plant sources constitute a promising pipeline to find new antimalarial molecules [2]. In a previous study, we explored the antiplasmodial properties of ellagic acid (EA), a molecule recently identified during bio-guided fractionation of West African plants used in traditional medicine to treat malaria crises [3].

EA is widely known for its antioxidant properties on diverse cellular models though the mechanisms of action are unknown [4]. EA has also shown high antiplasmodial activities *in vitro* (IC_50_ values ranging from 105 to 330 nM whatever the chemosensitivity of the strains tested) and *in vivo* (ED_50_ value by the intraperitoneal route inferior to 1 mg/kg/day) with interesting selectivity and therapeutic indices [3]. We previously demonstrated that the maximum antiplasmodial action of EA was at the trophozoïte and schizonte stages of the *Plasmodium* erythrocytic life-cycle [3]. These parasitic stages are known for their high amount of ferriprotoporphyrin IX (FP) produced during hemoglobin digestion [5]. FP induces oxidative damage to the parasite leading to its death and thus the *Plasmodium* needs to detoxify FP formation to remain viable. FP is detoxified by its biomineralization to hemozoin and degraded *via* glutathione (GSH) by a radical-mediated mechanism [6]. Furthermore, we initially showed that the known antioxidant N-acetyl-L-cysteine (NAC), that was expected to increase the cellular levels of GSH, diminished the antimalarial efficacy of EA *in vitro* [3]. NAC was also an antagonist to the antiplasmodial activity of chloroquine [6].

In this current study, we investigated the oxidative conditions inside *P. falciparum* to understand the mode of action of EA. To do this, we studied the antiplasmodial efficacy of EA at the trophozoïte and schizonte stages during which *Plasmodium* is subjected to important oxidative conditions. Our strategy was to explore the possible interactions between EA and FP leading to the preservation of the oxidative conditions that are able to kill the parasite. We measured the amount of intracellular reactive oxygen species (ROS) in *P. falciparum* after EA treatment, as well as the influence on its antiplasmodial activity of molecules such as antioxidants (NAC and ascorbic acid), an iron chelator (desferrioxamine) and an inhibitor of *de novo* glutathione synthesis (D,L-Buthionine-(S,R) sulphoximine (BSO)). Finally, we evaluated the effect of the glutathione content inside the parasite on the *in vitro* antimalarial action of EA.

## Results

### Ellagic Acid Treatment and the Amount of ROS in the *P. falciparum* FcB1 Strain at the Trophozoite and Schizonte Stages

**Figure 1:**
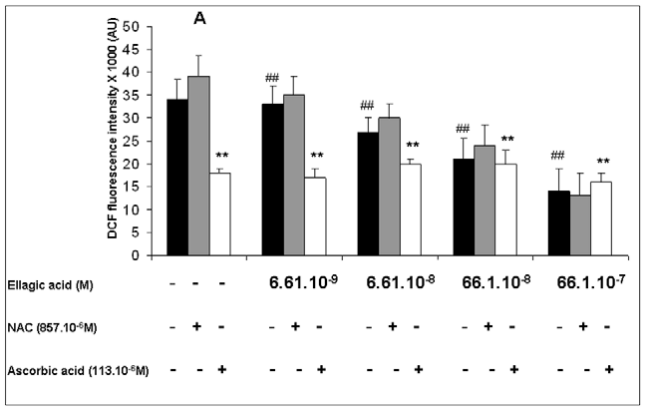
ROS generation in *Plasmodium falciparum* was detected by DCFDA probe. All parasitized erythrocytes were labeled with DCFDA for 30 minutes. After washing, they were incubated for 3 hours with or without ellagic acid, ascorbic acid and NAC, alone and in combination. The level of ROS in the parasites was detected by DCF-dependent measurements. The data represent the mean ±SEM of four different experiments with triplicate sets in each assay. In the first column (**A**), the fluorescence intensity in parasites without ellagic acid is represented. The black band (control value) corresponded to the fluorescence intensity in untreated parasites incubated 3 hours. The grey and white bands corresponded to the fluorescence intensity in parasites only treated with NAC (857.10^−6^ M) and ascorbic acid (113.10^−6^ M) respectively. In the next four columns, the black bands are the fluorescence of parasites after treatment with various concentrations (between 6.61×10^−9^ and 6610×10^−9^ M) ellagic acid alone. The grey and white bands correspond to the ellagic acid treatment with NAC and ascorbic acid respectively with the same concentrations as in the first column. **p<0.01 indicates a significant difference compared with the untreated parasites. The quantity amount of ROS was compared according to the increased of EA concentration, ## p<0.05 indicates the significant difference.

To evaluate the antioxidant properties of EA in *P. falciparum*, we analysed the intraerythrocytic presence of oxygen species and also the potential influence of antioxidant molecules such as NAC and ascorbic acid on the ROS production (Figure 1).

Our unpublished data confirmed that the ROS show a steady increase in the fluorescence intensity in the control wells without drugs, suggesting that ROS are formed spontaneously in the *Plasmodium* parasites; the maximum fluorescence being registered at 3 h.

EA induced a concentration-dependent decrease in the generation of ROS for the concentrations from 6.61 to 6610 nM (Figure 1). Whatever the EA concentration, N-acetyl-L-cysteine (NAC) did not influence the generation of ROS, whereas ascorbic acid alone significantly decreased it (p<0.05). The combination of EA with ascorbic acid had no potentiation effect on the ROS decrease in infected erythrocytes (Figure 1).

Rhodamine 123 staining conferred green fluorescence to mitochondria under fluorescence microscopy, confirming the viability of the parasites for the untreated controls but also for antioxidant-treated Plasmodium parasites. In parallel with the rhodamine 123 staining, one well with parasites treated under the same conditions but not stained were put again into continuous culture with RPMI plus 20% human serum. After 48 h in continuous culture, we observed growth development of the parasites by Giemsa staining. Whatever the conditions used (whatever the antioxidant concentrations used, alone or in combination), we confirmed the results obtained with the rhodamine 123 staining showing that all the parasites were alive since they were all able to grow and continue the normal erythrocytic cycles.

### Influence of NAC and Ascorbic Acid on the *in vitro* Antiplasmodial Activity of Ellagic Acid

The effects of ascorbic acid and NAC on the inhibition of parasite growth by EA were assessed on the *P. falciparum* strain FcM29. The concentrations of ascorbic acid (113.55 µM) and NAC (857 µM) chosen when used alone had no effect on the *in vitro* growth of the FcM29 strain of *P. falciparum* (data not shown). The antiplasmodial efficacy of EA was significantly decreased by ascorbic acid and NAC since the IC_50_ values of the combination of these molecules with EA were increased by 2.4 fold and 1.5 fold respectively (p<0.05) (Table 1). Ascorbic acid, which is more antioxidant than NAC (Figure 1), significantly reduced the antiplasmodial efficacy of EA compared with NAC that is less antioxidant.

**Table 1:** Inhibition of ellagic acid antiplasmodial activity by antioxidants (ascorbic acid, N-acetyl-L- cysteine and glutathione ethyl-ester) on the FcM29 strain of *Plasmodium falciparum*.

Compounds	Mean IC_50_ (nM) ± SD
**Ellagic acid**	97.15±16
**Ellagic acid + Ascorbic acid**	231.71±23**
**Ellagic acid + NAC**	149.66±8**
**Ascorbic acid**	>5.10^6^
**NAC**	>5.10^6^

Ascorbic acid (113.55 µM) and N-acetyl-L-cysteine (857 µM) had no effect on *in vitro Plasmodium* growth. Ascorbic acid and NAC significantly decreased (p<0.05) the ellagic acid antiplasmodial efficacy since the IC_50_ obtained were increased 2.4 fold and 1.5 fold respectively. Data are expressed as mean ±SEM for three independent experiments with triplicate sets in each assay.

** p<0.05 indicates a significant difference compared with ellagic acid mean IC_50._

### Effect of Ellagic Acid on the Reduced Glutathione Levels in the *Plasmodium falciparum* Strain FcM29-Cameroon

To ascertain whether FP was involved in the antiplasmodial action of EA, a synergistic test between ellagic acid and an iron chelator (desferrioxamine) was carried out. The modulating effect of desferrioxamine on the *in vitro* antiplasmodial efficacy of EA was additive in *P. falciparum* (Table 2). In combination with EA, BSO, an inhibitor of glutathione synthesis, also showed an additive effect with the strain FcM29 (Table 2). The *in vitro* efficacy of EA thus appeared to be linked to the GSH levels in *P. falciparum*.

**Table 2:** Effects of D,L-Buthionine- (S,R) sulphoximine (BSO) and desferrioxamine in combination with ellagic acid on the FcM29 strain.

Molecules tested with ellagic acid	Mean FIC_50_± SEM	Type of combination
**Desferrioxamine**	1.01±0.02	Additive
**BSO**	0.98±0.09	Additive

Values are the means of the FIC_50_ (which is an interaction coefficient indicating whether the combined effect of the drugs is synergistic, additive, or antagonistic) and standard deviations for assays run in triplicate on different days.

The combination was considered to be synergistic if the FIC_50_ was <1, additive if the FIC_50_ was equal to 1 and antagonistic if the FIC_50_ was >1.

As with glutathione-modulating drugs alone such as BSO, EA alone also significantly reduced the GSH levels (reduction of 50% and 57%, respectively) inside the parasite. However, the combination of EA and BSO decreased considerably further the GSH levels in the parasite with a strong synergistic effect (GSH content reduction of 93%) (Table 3).

**Table 3:** Determination of reduced glutathione in treated and untreated *P. falciparum.*

	Glutathione (nM/1.10^9^ parasites)	
	Concentration	Percentage (%)
**Control**	189±12	100
**IC_75_ BSO (224.91** **µM)**	95±7**	50
**IC_75_ Ellagic acid (1.3 µM)**	80±5**	43
**IC_75_ BSO + IC_75_ Ellagic acid**	13±1**	7

The GSH content was determined after 5 hours incubation of the asynchronized FcM29 strain of *Plasmodium falciparum* without drug addition (control) or with IC_75_ of BSO, IC_75_ of ellagic acid concentration or in combination of both drugs.

The glutathione contents of the controls were normalized to 100%. The GSH levels show percentages of treated parasites with reference to these basic GSH levels. Unsynchronized infected erythrocytes (15% parasitemia) were incubated for 5 hours. For this short-term incubation, we decided to apply concentrations of the respective drugs that led to 75% growth reduction in the 48 h growth inhibition assay and subsequently determined their glutathione content. IC_75_ values of both ellagic acid and BSO were 1.3 and 224.91 µMol respectively. The drugs were tested alone and in combination. The means ± SEM from three independent determinations are shown.

** p<0.01 indicates a significant difference compared with control parasites (untreated).

### *Ex-vivo* Short-term Cultures of *P. yoelii* with Ellagic Acid

To elucidate the impact of GSH levels on the *in vitro* antiplasmodial efficacy of EA, we investigated the *ex-vivo* activity of EA on a specific *P. yoelii* strain that has high intrinsic levels of GSH (26). The mean IC_50_ values of EA were significantly higher (4867±91 nM) on the *P. yoelii high GSH* strain than in the *P. yoelii* control (905.85±219 nM) (Table 4).

**Table 4:** *Ex-vivo* experiments: short-term culture of *P. yoelii* with ellagic acid.

Ex-vivo experiments	P. yoelii WT	«P. yoelii high GSH»
**Mean IC_50_ (nM)** ± **SD**	905.85±219	4867.34±9

Data are expressed as mean ±SEM for three independent experiments with triplicate sets in each assay.

The radioactive micromethod was used after 24 h of incubation at 37°C, to determine the IC_50_ of ellagic acid in the “*P.yoelii high GSH*” strain *versus* the controls corresponding to the wild type *P yoelii*.

## Discussion

The *Plasmodium*-infected erythrocyte is under constant oxidative stress. This is caused by exogenous reactive oxidant species (ROS) produced during the digestion of host cell haemoglobin [7].

Ellagic acid (EA) is a polyphenol known for its antiplasmodial properties [3] [8]–[9]. We previously demonstrated the suppressive effect of an antioxidant (NAC) on *in vitro* EA antiplasmodial activity [3] and supposed that the antiplasmodial action of EA was due to increased ROS generation, deleterious to *P. falciparum*. Furthermore, it had been suggested that the presence of metal ions such as iron surrounding polyphenols could significantly change their primary antioxidant activities to pro-oxidants [10]. In this context, the presence of FP (iron) from hemoglobin digestion inside the malaria parasite could change the properties of EA towards those of pro-oxidant molecules. Many antimalarial molecules, such as endoperoxides and quinolines, require oxidative conditions in the *Plasmodium* to act [11]. To the contrary, our results demonstrate that EA can have both antioxidant and antiplasmodial properties, whereas ascorbic acid, also an antioxidant (Figure 1), showed no *in vitro* antiplasmodial activity at the concentrations used (data not shown).

EA thus combined antioxidant activity and *in vitro* antiplasmodial activity against *P. falciparum*. These results suggest that the reduction of ROS by EA had no direct role in its antiplasmodial action. Desferrioxamine also has *in vitro* antimalarial action [12] and could induce an antioxidant effect [13]. This was verified by the combination of EA and desferrioxamine (iron chelator) being antiplasmodial and not antagonistic (Table 2).

It seems that the potential antioxidant action of EA against *P. falciparum* has no effect on its high *in vitro* antiplasmodial activity, and other mechanisms must be implicated. NAC also reduced the antiplasmodial potency of EA (Table 1) without an antioxidant effect on parasites compared with ascorbic acid (Figure 1). NAC is a well-known antioxidant that restores the GSH content inside cells. In this study, the duration of incubation of NAC with parasites could be too short to observe its antioxidant effect, whereas the GSH restoration by NAC could inhibit the antimalarial action of EA. To further clarify the role of cellular redox changes such as GSH depletion in EA-induced *Plasmodium* death, we determined the GSH content of the *Plasmodium* after treatment with several molecules. EA alone, as well as BSO alone, decreased the GSH content of *P. falciparum* (Table 3). The combination of both these drugs significantly decreased the GSH content compared with either drug alone (Table 3). This is the first time that the *in vitro* reduction of intraplasmodial GSH by EA treatment has been evoked. Another study only reported the depletion by BSO of the GSH content in *Plasmodium falciparum* [14].

EA is also a known inhibitor of human glutathione-S-transferase (GST) [15], and the inhibition of recombinant *PfGST* and *PfGR* by EA has been also demonstrated [16]. Taken together, all this data could suggest that GSH plays a role in the antimalarial properties of EA. GSH is one of the most well-studied intracellular compounds that plays a key role in cellular defenses against oxidative damage [17]. GSH synthesis is controlled by gamma-glutamylcysteine synthetase (GCS) and glutathione synthetase (GS) and is also regulated by glutathione-S-transferase (GST) and glutathione reductase (GR) [14]. The action of the enzymes GST and GR could be one of the potential pathways involved in the antiplasmodial efficacy of EA [16]. The enzyme GR, which is involved in antioxidant defense in *Plasmodium* species, has attracted particular attention as a potential therapeutic target. Biochemical studies have shown inhibition of *Plasmodium* both *in vitro* and *in vivo* after treatment with methylene blue, a molecule that reduced the GSH content in malaria parasites [18]–[19].

To confirm this possible mode of action for EA *via* GSH, *ex-vivo* assays were carried out with a selected *Plasmodium yoelii* strain that overproduces GSH compared with the parental wild type (WT) *P. yoelii* [19]. The results showed that the control parental *P. yoelii* strain was highly sensitive to EA compared with the *P. yoelii high GSH* line (Table 4). This result confirmed the potential negative influence of the *Plasmodium* GSH content on the antiplasmodial activity of EA.

In conclusion, EA is a natural compound with very high activity against malaria parasites without toxicity. The *in vitro* study of the antiplasmodial mechanism of EA demonstrated that oxidative conditions are involved and GSH metabolism seemed to be one of the targets of this molecule as is the case for some other known antimalarial drugs (quinolines and endoperoxides). Given its low toxicity, its ease of supply and its now known mechanism of action, EA appears as a promising drug candidate in the fight against malaria.

## Materials and Methods

### Compounds

Ellagic acid hydrate was obtained from Acros Organics (Belgium), molecular weight: 302.19 g/mol. D,L-Buthionine-(S,R) sulphoximine (BSO), ascorbic acid and N-acetyl-L-cysteine (NAC) were purchased from Sigma (France).

### Strains Used

The *P. falciparum* FCM29 strain (IC_50_ for chloroquine: 400 nM, IC_50_ for artemisinin: 3 nM) was used for *in vitro* experiments, *in vitro* potentiation tests and analysis of GSH levels. The FcB1 strain (IC_50_ for chloroquine: 110 nM, IC_50_ for artemisinin: 3.5 nM) presenting a Knobs+ phenotype was the best strain to obtain the synchronization of parasites in the trophozoite and schizonte stages. The parasites were then used to evaluate the antioxidant properties.

### *In vitro* Antiplasmodial Activity

These strains were continuously cultured using standard methods [20] with modifications [21]. The *in vitro* antiplasmodial activity was evaluated by the radioactive micro-method as previously described [22]. Each IC_50_ value was calculated as the concentration inhibiting 50% of parasitic growth [21].

### Analysis of Antioxidant Properties

Previously, we showed that the maximum of EA activity *in vitro* was reached at the trophozoïte and schizonte stages of *P. falciparum* FcB1 strains [3]. These stages are known to correspond with the maximum production of ROS during oxidative metabolism of the parasites.

All the experiments were carried out during this phase of the parasite erythrocytic cycle. Strains FcB1-Colombia were synchronized to trophozoite and schizonte stages. The method consisted of alternatively synchronizing young forms with 5% d-sorbitol and late forms with Plasmion followed by a 50–90% Percoll gradient centrifugation [23] to obtain a high concentration of trophozoite and schizonte stages.The trophozoite and schizonte-infected erythrocytes obtained were used in the determination of the antioxidant properties of EA. Measurements were monitored using dichlorofluorescein diacetate (DCF-DA) (Sigma, France). This assay was based on the oxidation of DCFH to the highly fluorescent dichlorofluorescein (DCF) [24].

The fluorescence probe was added to the synchronized parasitized erythrocytes (3.10^5^ cells/ml) at a final concentration of 20 µM. After 30 minutes of incubation of the cells with the DCF (2′-7′ dichlorofluorescein) and washing, the pre-labeled cells were incubated with concentrations of EA, ascorbic acid and NAC alone or in combination for 3 h at 37°C in the dark. The fluorescence of DCF was measured using a Perkin-Elmer LS-50B spectrofluorimeter with excitation and emission wavelengths set at 480 and 539 nm, respectively. EA, ascorbic acid and NAC were taken from 1 mg/ml stock solutions and added to the suspension of parasitized erythrocytes at concentrations in PBS (pH 7.4) in the range of 6.61 to 6610 nM for EA, 113.55 µM for ascorbic acid and 857 µM for NAC. The control cells were treated only with PBS.

### Determination of Parasite Viability after Treatment with Ellagic Acid

Coloration with rhodamine 123 (Fluka) was carried out to evaluate the viability of parasites treated with high concentrations of EA after 3 hours of incubation [25]. Rhodamine 123 (R123) stain confers green fluorescence to mitochondria, only if the parasites are alive (polarized mitochondria). Staining was done with 20 µl of infected erythrocytes for 5 min at 37°C (0.5 µg/ml R123 in RPMI) then infected erythrocytes were washed twice with RPMI and resuspended in 6 ml of RPMI supplemented with 5% human serum (EFS, French blood bank) and incubated for 30 min as in standard culture conditions. The stained thin blood smears were examined at 100 × magnification with an epifluorescence microscope and the viability of the parasites in the infected erythrocytes was calculated as a percentage of the relevant control.

### *In vitro* Potentiation Tests

The synergy between EA and BSO or desferrioxamine was assessed by potentiation experiments as previously described [26]. The chloroquine-resistant *P. falciparum* strain FcM29-Cameroon (IC_50_ for chloroquine of 400 nM) was used. Several combinations of EA and the other test molecules were incubated in 96-well plates, and the inhibition of parasite growth was evaluated as described above. The EA 50% fractional inhibition concentrations (FIC_50_s) were calculated by dividing the IC_50_ of the combination by the IC_50_ of EA alone and the IC_50_ values of the other molecules were also calculated. Potentiation results are isobolograms constructed by the fractional inhibition concentrations indicating the type of the combined effect of the drugs. The final value of the FIC_50_ indicated if the interaction was additive (FIC_50_ equal to 1), antagonistic (FIC_50_ was >1) or synergistic (FIC_50_ was <1).

### *Ex*-*vivo* Chemosensitivity Test

We possess a special murine *P. yoelii* strain, namely “*P. yoelii high GSH*”, which detoxifies free heme differently from the hemozoin formation. Indeed, one of the characteristics of these *P. yoelii* lines is that the hemozoin content is considerably decreased, while the GSH level is increased [19]. The parasitized blood samples were collected by retro-orbital puncture on anesthetized infected mice. After the removal of leucocytes, the infected erythrocytes were further washed twice with PBS and then plated out with various concentrations of EA. The radioactive micromethod was used to determine the IC_50_ values of EA on this *ex-vivo* “*P. yoelii high GSH*” strain *versus* the controls corresponding to the parent *P yoelii* line.

### Animal Procedures

All procedures involving living animals were carried out in the animal facilities of the Parasitology Department of the Toulouse (France) University Hospital under the control of the National Veterinary Services and performed according to European regulations (EEC directive 86/609 dated 24 November 1986). The staff in charge of the animal experiments had received the appropriate training. All *in vivo* studies were approved by the French Institutional Animal Experimentation Ethics Committee (approvals MP/R/05/32/11/07 for the selection of drug resistant clones).

### Determination of Plasmodial Reduced Glutathione (GSH)

The determination of plasmodial reduced GSH in untreated and treated erythrocytes infected with *P. falciparum* FcM29 strains, with the concentration corresponding to the IC_75_ value of EA (1.3 µM) and BSO (224.91 µM) was carried out. Erythrocytes were incubated for 5 hours to determine the effect of the drugs on the GSH levels after short-term incubation. The total plasmodial GSH content of both treated and untreated infected erythrocytes was determined as described by Luersen et al. [27]. Briefly, the infected erythrocytes (around 10% parasitemia) were incubated for 5 minutes at 4°C in PBS containing 0.05% saponin, to lyse the infected red blood cells After centrifugation (1500 g for 5 minutes), the supernatant was removed and the parasites recovered in the pellet were washed twice in ice cold PBS. After another lysis process (the suspension was submitted to 3 freeze/thaw cycles using liquid nitrogen and a 37°C water bath), two volumes of 5% sulphosalicylic acid (SSA) (Merck, Germany) were added to the parasites and mixed for 1 minute. The samples were centrifuged at 4°C for 15 minutes at 18000 g and the reduced GSH level determined in the supernatant with Ellman’s reagent: 5,5′-dithiobis (2-nitrobenzoic acid) (DTNB) (Sigma, France). 50 µl of supernatant was mixed with 150 µl of Tris-HCl/EDTA buffer pH 9 and 10% DTNB solution in DMSO (dimethylsulfoxide) (Sigma, France). The formation of thionitrobenzene (TNB) (yellow product) was measured spectrophotometrically at 405 nm. A standard curve was constructed with serial GSH (Sigma) dilutions in 5% SSA.

### Statistical Analysis

All experiments were reproduced at least three times and results are given as mean +/− SEM. Amounts were compared by the Fisher’s exact test and the significance was indicated as follow: p<0.05. All tests were performed using the SigmaStat (2.03) statistical program (SigmaStat, Heame Scientific Software, Chicago, USA).
